# Digital Ischemia in Scleroderma Spectrum of Diseases

**DOI:** 10.1155/2010/923743

**Published:** 2010-08-31

**Authors:** Elena Schiopu, Ann J. Impens, Kristine Phillips

**Affiliations:** Division of Rheumatology, University of Michigan Scleroderma Program, 24 Frank Lloyd Wright Drive, Ann Arbor, MI 48106, USA

## Abstract

Systemic Sclerosis (Scleroderma, SSc) is a disease of unknown etiology characterized by widespread vasculopathy and extracellular matrix deposition leading to fibrosis and autoimmune processes. Digital ischemia (digital ulcers (DUs)) is the hallmark of SSc-related vasculopathy and is characterized by endothelial dysfunction leading to intimal proliferation and thrombosis. It happens frequently (30% of the patients each year) and it is associated with significant morbidity. This paper summarizes the current information regarding pathogenesis, definitions, management, and exploratory therapies in DUs associated with SSc.

## 1. Introduction

The subject of digital ischemia in systemic sclerosis is complex and poses various challenges in terms of diagnosis, classification, risk factors, therapy, and morbidity. Raynaud's phenomenon (RP), characterized by exaggerated but reversible vasospasm in response to cold or stress, is the presenting symptom in over 90% of the patients with SSc [1]. RP is the most common manifestation of the SSc-related endothelial dysfunction and digital ulcers (DUs) are a clinical manifestation of SSc-related vasculopathy.

 Digital ulcers in SSc are defined as necrotic lesions occurring at the distal aspects of fingers or toes. The underlying phenomenon is compromise of the arterial lumen which occurs as a combination of 2 major contributing factors:

1. vascular wall structural (intimal proliferation) and functional (overproduction of vasoconstrictors) abnormalities,
2. a variable degree of intraluminal thrombosis.

DUs are painful, heal slowly, and lead to a great deal of disability. Due to the inadequate blood supply and break in the skin, the ischemic lesions are prone to infection, loss of digital tissue, and progression to gangrene that requires amputation. 

Currently, there is no official algorithm for diagnosis and therapy of digital ischemia in SSc. A conventional therapeutic approach to digital lesions should include vasoactive medications, antiplatelet agents, antibiotics as needed, and analgesia. The response to vasodilators in patients with SSc is variable and often disappointing. There is a visible need for strategies to facilitate healing of the DUs and to prevent occurrence of new ones.

## 2. Definition of Digital Ulcers in SSc

The correct diagnosis of DUs is instrumental both in clinical practice and in clinical trials focused on digital ischemia. Almost all SSc patients experience involvement of their hands: ischemic lesions, local infection, calcinosis, and traumatic ulcers occurring in areas affected by flexion contractions. Although the SSc-related vasculopathy affects the healing time of all the acral lesions, it is crucial to clinically define the true ischemic lesion. 

A recent study tested the intra- and interobserver variability in defining DUs among clinicians with an interest in scleroderma [2]. 50 individuals (mostly rheumatologists) were shown pictures of various hand lesions and were asked to qualify the lesions (“ulcer” versus “no ulcer”) and if “ulcer”, to quantify it as “active” or “inactive”. Although the intrarater reliability was high (average kappa value of 0.81), the interrater reliability was poor (kappa coefficient of 0.46), so individual examiners were consistent with their assessment, while different examiners disagreed. This lack of agreement among rheumatologists who evaluate digital lesions on a regular basis may have an impact on interpretation of the results of clinical studies and more so on initiating and maintaining treatment of DUs in clinical practice. 

**Figure 1:**
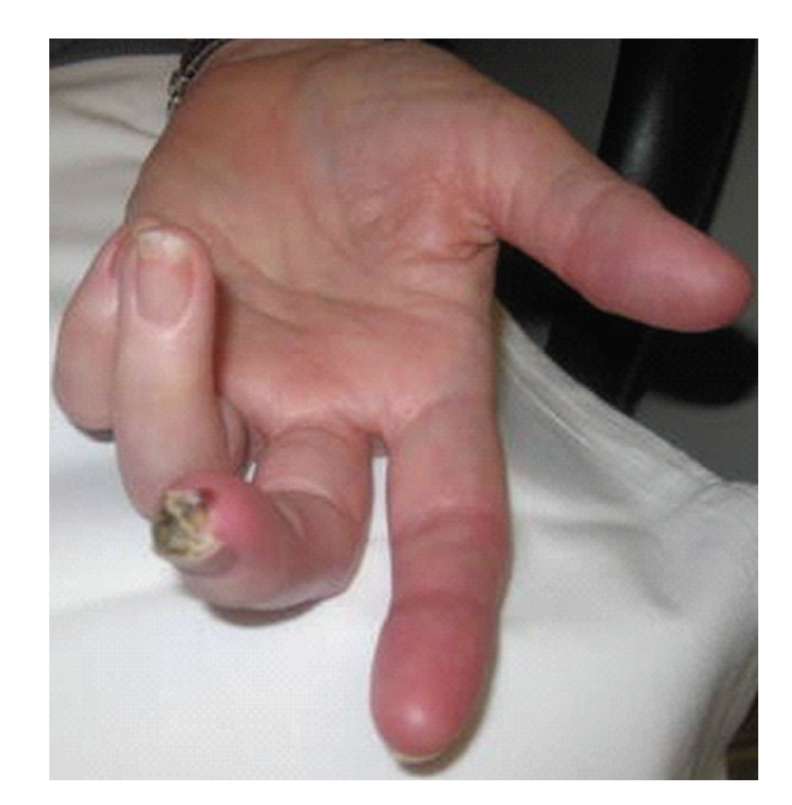
True digital ulcer.

**Figure 2:**
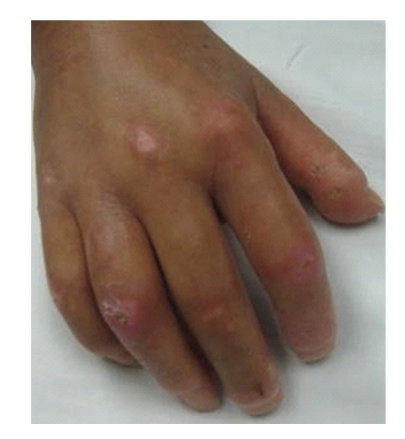
Traumatic ulcer.

One of the more precise definitions of SSc-DUs was described in the RAPIDS-1 clinical trial [3]. The definition was based on expert consensus and is currently used in the majority of trials focused on DUs. Digital ulcers are defined as a denuded area with well demarcated borders, involving loss of dermis and epidermis. They are located on the volar surface of the fingers, distal to the proximal interphalangeal joints (Figure 1). The DUs do not occur in the interphalangeal creases and should not be confused with paronychia, areas with underlining calcinosis, or traumatic lesions located on the dorsum of the hands (PIPs or MCPs) (Figure 2). A recent article focused on definitions and subclasses of SSc-DUs (1614 digital lesions were prospectively observed over 4 years) [4]. The digital lesions were classified as digital pitting scars (DPSs), DUs, calcinosis, and gangrene. Clinical characteristics, depth (superficial, intermediate, and deep) and time to healing of the lesions were recorded. The overwhelming majority of digital lesions, were DUs and DPSs (92.7%). The digital lesions were located more frequently on the second and third digit and mostly on the fingertip area. Presence of calcinosis, wet or dry necrosis, and infection significantly delayed the time to healing. In this study, the definition used for the “pure” DUs matched the one from the RAPIDS studies and the DUs had a distinct natural history. The authors concluded that a precise classification of the subtype of digital lesion is important when different therapies are entertained: DUs due to calcinosis may not be as responsive to vasodilators as a purely ischemic DUs would be.

## 3. Pathogenesis of Digital Ulcers in SSc

The initial trigger in SSc-related vasculopathy is unknown. It is believed that smooth muscle cells migrate into the intimal layer of the microvasculature and differentiate into myofibroblasts that secrete collagen and an other extracellular matrix. This process leads to a fixed narrowing of the intravascular lumen which hinders the blood flow and causes chronic tissue ischemia. Histological studies showed that 18 (79%) of the 23 evaluable biopsies of digital arteries of patients with SSc had greater than 75% luminal narrowing [5]. 

 Aside from the structural change, the endothelial cells are perturbed, possibly through ischemia-reperfusion injury or an autoimmune insult [6] leading to an increased production of vasoconstrictors such as endothelin and an underproduction of vasodilators such as prostacyclin and nitric oxide. Another proposed mechanism of endothelial injury is the presence of antiendothelial cell antibodies [7]. One other possible consequence of the endothelial damage is platelet activation with release of thromboxane [8] which leads to intraluminal thrombosis.

## 4. Natural History of Digital Ulcers in SSc: Incidence, Risk Factors, and Clinical Impact

**Figure 3:**
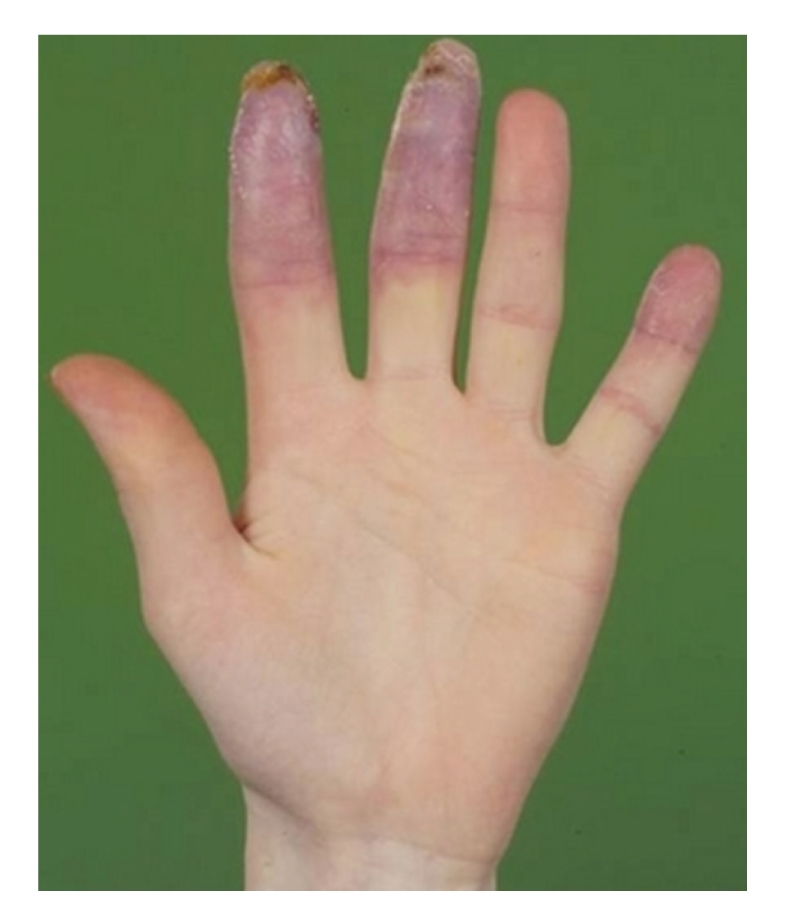
Ischemically threatened digit.

Various studies have revealed that 15%–25% of SSc patients have active DUs [9] and 35%–50% had a history of DUs [10]. DUs are painful, disabling, and associated with a variable rate of progression to gangrene or infection leading to amputation. A particularly emergent situation is the ischemically threatened digit (Figure 3) due to the high rate of need for surgical intervention. 

Although a prospective registry of patients with SSc-DUs is lacking, the available data from retrospective analysis [11, 12] outline the following risk factors for developing DUs:

1. male sex,
2. presence of pulmonary hypertension and/or lower DLCO,
3. diffuse subset of the disease,
4. early onset of SSc,
5. presence of antitopoisomerase I antibodies (anti-topo I),
6. smoking.

Patients with DUs developed internal organ involvement 2-3 years earlier compared to patients without ulcers [12]. 

The use of nailfold videocapillaroscopy (NVC) may be a novel tool useful to predict development of SSc-DUs: the specificity and sensitivity were as high as 85.9% and 94.3%, respectively [13]. 

 Episodes of DUs tend to reoccur, with 66% of patients having more than one episode and 50% having more than 2 episodes over a period of 7.26 years [14] despite the use of vasodilators. In the same cohort, the ulcers occurred more frequently in the second and third digits (II: 32.5%, III: 32.5%) and were equally distributed among both hands. Thirty patients (67%) had critical finger ischemia at least once, and 43% of patients received at least one course of intravenous iloprost; 7% of patients underwent surgical amputation. 

 The morbidity related to presence of DUs is significant. In the Pittsburgh database [15], the disability measured by the Scleroderma HAQ (SHAQ) was significantly greater in patients with persistent DUs. Patients also had more hospitalizations and more hospitalizations for antibiotics than patients without DUs (16 versus 9%). The incidence of gangrenous lesions was 11% and increased with the length of time since the first DUs, especially after 4 years. Data from the Randomized Placebo-Controlled Study on Prevention of DUs in SSc (RAPIDS-2) trial conducted in 188 patients with active DUs reported the incidence of amputation to be 11% (1-2%/patient-year of followup) [16]. 

A retrospective analysis of all the hospitalized cases due to ischemically threatened digits (ITDs) over a period of 10 years in a tertiary care center identified 79 patients, of which 22.8% had SSc [17]. In that particular cohort, the rate of amputation was 48.1%, and male sex, a history of previous ITD, or ITD developed in the hospital were associated with a higher rate of amputation.

## 5. Therapeutic Options for SSc-Digital Ulcers

The therapeutic approach in SSc-DUs poses multiple challenges. Ischemic DUs can be confused with DUs due to trauma or calcinosis. There is disagreement about the difference between active and nonactive ulcers. The pathogenesis of SSc-DUs is complex, and it involves multiple pathways which need to be addressed concomitantly. Pain and infection are common comorbidities that require supportive therapy. Response to the instituted medical therapy needs to be continuously assessed to detect the need for additional drug therapies or to consider surgical options. 

The available clinical trials for treatment of active SSc-DUs, although largely negative, have contributed to our knowledge about outcome measures in this disease [18]. The crude measurement of the depth and length of DUs is not feasible due to the location of the ulcerations and the associated pain. The only direct parameter remains the absolute healing of the DUs, which includes an anatomical (re-epithelialization of the area) and a physiological (pain cessation) component. Other indirect parameters, like instruments focusing on quality of life (HAQ) and function of the hand (UKFS and the Michigan Hand Questionnaire) require further validation studies. 

The DUs therapeutic approach includes: supportive measures, pharmacological interventions, and surgical options. 

### 5.1. Supportive Therapies

Patients who develop SSc-DUs must be educated to keep their whole body warm (not just the hands) and to avoid direct trauma to the tips of the digits. It is paramount for smokers to quit. All other vasoconstrictors (cocaine, sympathomimetics) should be discontinued. 

Pain related to SSc-DUs is exquisite and lasts as long as the DUs is active, which could be months. The intensity of the DUs-related pain is significant and could lead to anxiety which potentially worsens the Raynaud's symptoms which in turn could contribute to a lengthier healing process. Pain management should be instituted promptly and adequately escalated. Although the nonsteroidal anti-inflammatory drugs are very efficient, they should be avoided in favor of acetaminophen or opiates due to their vascular side effects. Since the cause of the pain is tissue ischemia, the real solution is improving the oxygen delivery to the affected area. 

Infections are common in SSc-DUs and heal slowly because of the poor circulation. Clinicians should inspect each ulcer carefully at each visit. Clinical clues to DUs infection are an increase in the amount of pain (and a change of character to throbbing) and presence of purulent discharge. Simple gram positive coverage is usually very efficient but broad coverage antibiotics might be used if the ulcers are more extensive. Patients may require more than one course of antibiotics. 

If osteomyelitis is suspected, patients should be managed by a multidisciplinary team that includes infectious disease and orthopedic specialists. Intravenous antibiotics, hyperbaric therapy, and surgical amputation may be helpful.

### 5.2. Pharmacological Therapies

The purpose of the DUs treatment is to reduce their overall burden and impact on quality of life. Aside from controlling the pain and preventing infections, clinicians need to restore the hand function, improve the digital circulation, and promote healing of the existing DUs while preventing formation of new ulcers. Despite the substantial impact that SSc-DUs have on function and quality of life, there is currently no accepted therapy algorithm nor any FDA- approved therapies. 

The treatment algorithm should mirror the pathogenesis of SSc-DUs and should include antiplatelet agents and vasoactive agents. It has become common practice that patients with a history of DUs or an active DUs should take at least a low dose of aspirin (81 mg) daily. Clopidrogrel is being used with or without aspirin for active DUs but there are no published data on safety and overall efficacy. 

Vasoactive therapy includes the background therapy for Raynaud's Phenomenon (RP) and agents from a few available classes that were shown to improve SSc-DUs or are undergoing experimental trials. Potential therapies for SSc-DUs will be outlined below by class.

#### 5.2.1. Calcium Channel Blockers

Calcium channel blockers (CCBs) are widely used for the treatment of primary and secondary RP, reducing the severity of attacks by 35% [19]. The effect of CCB on SSc-DUs was reported in a small 16-week randomized controlled trial that compared oral nifedipine with intravenous iloprost: the mean number of ulcers decreased (from 4.3 to 1.4) but there were no physiological changes in the microvasculatory blood flow or the temperature of the hands [20].

#### 5.2.2. Phosphodiesterase-5 Inhibitors

Phosphodiesterase-5 inhibitors (PDE5 inhibitors) induce vasodilatation by increasing the levels of available endogenous nitric oxide (NO). The positive effect of sildenafil on RP was shown in a randomized controlled crossover study [21] and case reports and series indicate the benefit of sildenafil in SSc-DUs [22, 23]. An open label pilot study using a maximum dose of sildenafil in 19 patients with SSc-DUs showed a total reduction of ulcers from 49 to 17 (*P* < .001) after 6 months [24]. 

Shenoy et al. presented SSc-DUs healing results in a crossover trial of tadalafil [25]. The RP improvement was clinically significant, with a surprising absence of placebo effect. This is contradicted by another randomized crossover trial of tadalafil in SSc-RP which showed no benefit of the drug over placebo [26]. 

The role of PDE5 inhibitors in SSc-DUs needs to be further evaluated in prospective, randomized trials.

#### 5.2.3. Endothelin Receptor Antagonists

The endothelial injury that is the hallmark of ischemic digital ulcers corresponds with an increase in levels of endothelin 1 (ET-1) [27, 28]. ET-1 is a potent vasoconstrictor that mediates its actions through two different receptors: endothelin type A (ET-A) receptors are found on vascular smooth muscle cells while endothelin type B (ET-B) receptors are found on both endothelial cells and vascular smooth muscle cells. 

Bosentan is a receptor antagonist with activity against both types of receptors, ET-A and ET-B. Anecdotal reports and case series have suggested that bosentan may be helpful in reducing ulcer size or improving healing [29–31]. Other studies have demonstrated an improvement in flow-mediated dilatation of microvasculature with bosentan therapy in patients with systemic sclerosis [32]. 

The RAPIDS-1 trial analyzed the effect of Bosentan on the prevention and treatment of existing digital ulcers [3]. This randomized, placebo-controlled study of 122 patients with systemic sclerosis and preexisting digital ulcers evaluated a primary outcome of bosentan on number of new digital ulcers during a 16-week study period. Patients were treated with bosentan 62.5 mg twice daily for four weeks then 125 mg twice daily for 12 weeks. While no significant difference was seen in healing of existing ulcers, there were significant differences in hand function and number of new ulcers in the treatment group (1.4 versus 2.7). 

In the open-label extension of the study 88 patients continued bosentan for 12 additional weeks (57 of these patients were previously treated with bosentan for 16 weeks and so received a total of 28 weeks of treatment) [33]. At the end of the extension period, 65% of patients did not develop any new digital ulcers while 8% (7 patients) developed more than two new ulcers. The mean number of new DUs after 12 weeks of open-label therapy was 0.7 (all subjects), 0.5 (subjects previously on placebo), and 0.8 (previously on bosentan). RAPIDS-2 was designed to confirm the positive effects of bosentan at reducing new DUs in 188 patients with at least an active SSc-DUs over a variable treatment course [34]. Patients received 62.5 mg bosentan twice daily for 4 weeks then 125 mg twice daily for 20 to 32 weeks. None of the measures of digital ulcer healing differed between the two groups (time to healing of a selected cardinal ulcer, time to healing of all digital ulcers, and percent of patients with complete healing). However, the total number of new ulcers during a 24-week followup period was 1.9 on bosentan and 2.7 on placebo (*P* = .035). Unlike what is known of RP, there was no clear influence of season on SSc-DUs, indicating that DUs may be more related to the severity of vasculopathy. 

Overall, oral bosentan at a dose of 125 mg twice daily had no effect on ulcer healing in patients with digital ulcers and SSc. Although bosentan seemed to have lessened the ulcer burden in patients with more than four concomitant ulcers, it had no effect on RP. 

There are also anecdotal reports and case reports of combination treatment with bosentan [35]. This case report of a 73-year-old patient with systemic sclerosis reveals that she was treated with both sildenafil and bosentan which resulted in complete healing of the digital ulcers. 

Sitaxentan is a selective endothelin type A receptor antagonist that has been reported to help heal SSc-DUs in case reports [36], but no randomized clinical trials are ongoing.

#### 5.2.4. Prostacyclin Analogues

Prostacyclins are potent vasodilators that also inhibit platelet aggregation and smooth muscle proliferation in the blood vessels walls. Various forms of prostacyclins are approved for use in idiopathic and connective tissue-related pulmonary arterial hypertension (PAH). 

Epoprostenol is an intravenous prostanoid. In the pivotal randomized controlled trial of epoprostenol in patients with SSc-related PAH, the patients on epoprostenol had 50% fewer new DUs than those treated with conventional therapy, although no effect on healing of the existing DUs was reported [37]. 

Iloprost is the most studied prostacyclin analog and is available in intravenous (IV), oral, and inhaled formulations. Outside the USA, cyclic use of intravenous iloprost is the standard of care for treatment of ischemically threatened digits and severe SSc-DUs. The most important clinical trial supporting the use of iloprost was a 9-week, double-blind placebo-controlled multicenter trial in patients with SSc-RP [38]. In this trial, the intervention consisted of IV iloprost 6-hours infusion (dose of 0.5–2 ng/kg per minute) for 5 days. A significant proportion of the patients receiving the active drug had at least 50% reduction in the number of DUs. Interestingly, although the DUs healing was noted at earlier time points, the effect persisted at 9 weeks, suggesting a potential “reset” effect that iloprost might have on the endothelium. In this particular trial, a trend towards prevention of new DUs was noted. A randomized controlled trial of oral iloprost at a fixed dose for patients with SSc-RP had negative results [39], most likely because there was no titration to the maximum tolerated dose. Higher doses of iloprost may be more effective in SSc-RP at the expense of increased side effects [40]. 

Oral beraprost, another available prostacyclin analog, was evaluated in a randomized controlled trial for SSc-RP [41] and it showed a trend towards fewer new DUs in the treatment group but no effect of the RP. 

Treprostinil is available in IV, subcutaneous, inhaled, and oral formulations. A pilot study of subcutaneous treprostinil in patients with SSc-DUs over a 12-week treatment period showed both healing and prevention of the DUs but only a few subjects were able to tolerate the injection site pain [42]. Treprostinil diethanolamine (TDE-SR) is an innovative salt form of treprostinil for oral delivery as a sustained release osmotic tablet for twice daily dosing. The pharmacokinetic profile of TDE-SR in patients with SSc-DUs is comparable to that of healthy controls [43] despite a variable degree of absorption related to SSc. An ongoing phase II randomized, double blinded, multicenter clinical trial of oral treprostinil in SSc-DUs is currently recruiting.

#### 5.2.5. Statins

It is well recognized that statins (3-Hydroxy-3-methylglutaryl-coenzyme A reductase inhibitors) have beneficial effects on ischemic vascular events and consequences of vascular injury [44]. An open-label study of 14 SSc patients showed that 12 weeks of atorvastatin improved the Raynaud's severity and prevented new SSc-DUs [44]. A large randomized, double-blinded, placebo controlled study of 84 patients with SSc and history of DUs showed a significant difference in the number of new DUs in the treatment group compared to placebo over 4 months (1.6 versus 2.5) [45]. Significant improvement in the Scleroderma Health Assessment Questionnaire Disability Index (SHAQ-DI) score and endothelial markers from the baseline was noted.

#### 5.2.6. Angiotensin-Converting Enzyme Inhibitors (ACEIs) and Angiotensin Receptor Blockers (ARBs)

The role of ACEI in the management of scleroderma renal crisis has changed the primary cause of mortality in SSc from renal to pulmonary complications. ACE inhibition counteracts the renin-driven hypertension but it also improves endothelial function and promotes vascular remodeling in chronic hypertension. 

A study of the long-acting ACEI, quinapril, used the rate of occurrence of new DUs in patients with SSc as primary outcome [46]. This was a multicenter, randomized, double-blind, placebo-controlled study of quinapril at 20 mg daily for at least 2 years but not more than 3 years. Of the 213 patients enrolled, 109 patients were followed for the full 3 years. At the end of the study there was no difference between the treatment and the placebo group in the number of new DUs, the total number of DUs, and measurements of RP (frequency and severity). As the authors commented, the lack of therapeutic effect was not attributable to insufficient statistical power. 

In a randomized 12-week trial comparing nifedipine to losartan, an ARB, in patients with primary and secondary RP (total number of patients of 52), losartan significantly reduced the RP severity and frequency [47]. This study focused mainly on RPs and there was no mention of SSc-DUs.

#### 5.2.7. N-Acetylcysteine

N-acetylcysteine (NAC) is the precursor of a major antioxidant, glutathione, and may have beneficial effects in SSc-DUs due to its vasodilating properties and impact on platelet aggregation. A pilot study of intravenous NAC in 20 patients with SSc-DUs showed that more than half of the DUs present at baseline completely healed after the 5-day infusion [48]. A prospective observational study of intravenous NAC dosed at 15 mg/kg/hr for 5 hours every 14 days was recently reported [49]. The median treatment was 3 years, and the mean of ulcers/patient/year decreased significantly from 4.5 to 0.81 with minimal reported side effects. Although promising, in order to better establish its use in SSc-DUs, this agent should be evaluated in a prospective, placebo controlled, randomized fashion.

#### 5.2.8. Vitamin E Gel

Vitamin E has been used for chronic cutaneous lesion and ulceration treatment based on its antioxidant and anti-inflammatory effects. An open-label study of 27 patients with SSc-DUs randomly assigned 15 subjects to vitamin E gel. Patients treated with vitamin E experienced significantly reduced healing time (13.2 versus 20.0 weeks, *P* < .0001) when compared to controls and improved pain resolution (*P* = .0022).

### 5.3. Surgical Options

Surgical options are reserved for treatment of severe or recurrent DUs that are recalcitrant to medical therapy. Available procedures are microsurgical revascularization of the hand and digital sympathectomy [50]. Since the size of the blood vessels involved in the pathogenesis of SSc-DUs is usually small, revascularization is not readily applicable. 

Sympathectomies are aiming to block the sympathetic nerve-mediated vasospasm which is thought to have an important role in digital ischemia. The long-term results of cervical sympathectomy have been discouraging. Local digital sympathectomy has the advantage of interrupting the sympathetic fiber more distally. During digital sympathectomy, the adventitia is excised, removing sympathetic fibers contained in the adventitia and most likely, the media. This procedure has been shown to help healing of SSc-DUs, improvement in pain, and prevention of new DUs for a mean of 31 months after the surgery [51]. The results of digital sympathectomies in patients with connective tissue disorders may not be as favorable as in patients with other diagnoses [52]. 

 Although the microvascular involvement in SSc is well known, there are reports of macrovascular involvement as well. A small study of 8 patients with DUs or gangrene related to SSc found macrovascular involvement of the ulnar artery (3 patients) and radial artery (1 patient) by arteriography [53]. A larger retrospective study identified 12 (63.2%) of 19 patients with SSc who underwent brachial arteriography to have ulnar artery involvement (occlusion and/or stenosis) [54]. Potential for revascularization in selected SSc patients with arteriographic evidence of ulnar involvement was shown in a retrospective chart review [55]: 15 patients with SSc-DUs and ulnar artery occlusive disease confirmed by angiography were reviewed. Eight patients who underwent ulnar revascularization combined with digital sympathectomy had improvement in the healing of the ulcers. 

The current evidence is limited but there is a role for brachial arteriography in selected patients with SSc-DUs to diagnose macrovascular involvement. More interventional trials are needed to assess the efficacy of ulnar revascularization in SSc-DUs prevention and treatment.

## 6. Conclusions

Digital ulcers affect patients with SSc with a frequency of 30% per year. A precise definition of the SSc-DU would be useful for the research community and also in clinical practice. The effect on function and quality of life of the SSc-DU is significant, so therapies focused on rapid healing and prevention of new ulcers are needed. Although patients note reduced pain and improved function when exposed to parenteral agents like iloprost, the more subtle agents that seem to be better at prevention (e.g., bosentan) offer very little benefit on pain and quality of life. Important clinical trials of oral or topical drugs with overall burden of the SSc-DUs as main outcome measure are ongoing.
